# A functional variant of *SMAD4* enhances macrophage recruitment and inflammatory response via TGF-β signal activation in Thoracic aortic aneurysm and dissection

**DOI:** 10.18632/aging.101662

**Published:** 2018-12-07

**Authors:** Ying Wang, Pei Yin, Yi-Huan Chen, Yun-Sheng Yu, Wen-Xue Ye, Hao-Yue Huang, Zhen-Chun Ji, Zhen-Ya Shen

**Affiliations:** 1 Department of Cardiovascular Surgery of the First Affiliated Hospital and Institute for Cardiovascular Science, Soochow University, Suzhou, Jiangsu, China; 2 Department of Thoracic Surgery, Taizhou People's Hospital, Taizhou, Jiangsu, China; * Equal contribution

**Keywords:** SMAD4, polymorphism, MØ recruitment, inflammatory, TGF-β, thoracic aortic aneurysm and dissection

## Abstract

Thoracic aortic aneurysm and dissection (TAAD) is the most fatal macro vascular disease. The mortality of 48h after diagnosis of dissection is up to approximately 50-68%. However, the genetic factors and potential mechanism underlying sporadic TAAD remain largely unknown. Our previous study suggested rs12455792 variant of *SMAD4* gene significantly contributed to the increased risk and might participated the pathological progression of TAAD. This investigation aims to test (1) the associations between rs12455792 and MØ recruitment, inflammatory response in aggressiveness of TAAD, and (2) the molecular mechanism accounting for their effects. In TGF-β signaling molecular detection, rs12455792 C>T variant activated the canonical and non-canonical TGF-β mediators. It also increased the secretion of chemotactic factors of HASMCs. To confirm the impact of this change, we detected MØ recruitment and infiltration in HASMCs and aortic tissues of TAAD patients. We found that MØ recruitment in cells and tissues with rs12455792 variant genotypes was increased than that in wild type groups. Moreover, rs12455792 variant increased M1 type inflammatory response, which might contribute much to TAAD progression. To mimic the *SMAD4* suppression effect of rs12455792 in vivo, we constructed the *SMAD4* KD mouse. After induction with Ang II for 4w, the thoracic aorta dilatation and vascular remodeling were more serious than that of wild type group. In conclusion, rs12455792 increased MØ recruitment, M1 type inflammatory response via activated TGF-β signaling, and further promoted vascular remodeling and pathological progress of TAAD.

## Introduction

Aortic aneurysm and dissection is the most fatal macro vascular disease, accounting for over 152,000 new deaths in the United States per annum [1]. Related studies have shown that the pathological mechanism of thoracic aortic aneurysm and dissection (TAAD) involves macrophages (MØ) recruitment, inflammatory reactions and vascular remodeling [2]. MØ is a pivotal mediator involved in vascular remodeling and aneurysm dissection and rupture [3]. Increasing evidences revealed that MØ presented anti-inflammatory effects via secreting proinflammatory factors and chemokines in vascular injury region, thus initiated wound-healing and tissue remodeling [4]. MØ acquires distinct functional phenotypes via different polarization phenotypes-M1 or M2. The imbalance between M1 and M2 type MØ accelerated the progression or rupture of aortic aneurysms [3].

*SMAD4*, also known as DPC4 (Deleted in pancreatic carcinoma 4, DPC4), is located at 18q21.1 [5]. It encodes the unique co-Smad molecule in the TGF-β signaling pathway [6]. TGF-β/Smad signaling plays an important role in the pathophysiological processes of vascular disorders such as revascularization and injury repair for vessel wall [7]. Generous studies revealed that mutations of members in TGF-β/Smad signaling were causative for macro vascular disease, e.g. Marfan syndrom (MFS) or Loeys-Dietz syndrome (LDS) [8-10]. *SMAD4* mutations were proven causing thoracic aortopathy and vascular malformation [11-13] since it was essential in vascular development [6]. Furthermore, *SMAD4* haploinsufficiency resulted in aortic aneurysm exacerbation in a MFS mouse model [14]. Zhang et al. also demonstrated that *SMAD4* deficiency elevated MØ infiltration and initiated thoracic aortic aneurysm and dissection formation in mouse model [15]. However, the genetic effect and exact role of *SMAD4* in the pathogenesis of human thoracic aortic disorders is largely unknown.

At first, we screened 20 SNPs (nearest gene were FBN1, TGFBR1, SMAD4, TYW1, LINC02398 and so on) using recent Genome-wide associated studies (GWAS) based TAAD reports [16-18] and OMIM database. Then candidate SNPs were genotyped in 202 TAAD patients and 400 healthy controls. 5 significant SNPs were identified. Among them, rs12455792 (*SMAD4*) C>T variant significantly increased TAAD risk and correlated with increased aortic diameter. We further detected the impact of other 4 *SMAD4* SNPs on TAAD risk, and the potential impact of rs12455792 on *SMAD4* expression and cell function [19]. But the potential mechanism between rs12455792 variant, MØ infiltration, vascular remodeling and pathological progression of TAAD remained unclear.

Here we identified 5 significant SNPs on the basis of GWAS-based TAAD reports. Among them, rs12455792 was correlated with aortic diameter of patients. We further explored whether rs12455792 contributed to the MØ recruitment, vascular remodeling and TAAD progression. To mimic the impact of rs12455792 - *SMAD4* low expression generally, we constructed the *SMAD4* KD mouse and detected the associated pathological progress of thoracic aortic aneurysm and dissection. Our study elucidated the potential mechanism for differences in susceptibility and prognosis of TAAD between patients with CC, CT or TT genotypes. These novel findings may shed light on the role of rs12455792 and *SMAD4* in pathogenesis of TAAD and provide a predictive marker for optimizing clinical trial design and individualizing therapeutic plans.

## RESULTS

### Genetic association studies

**Figure 1:**
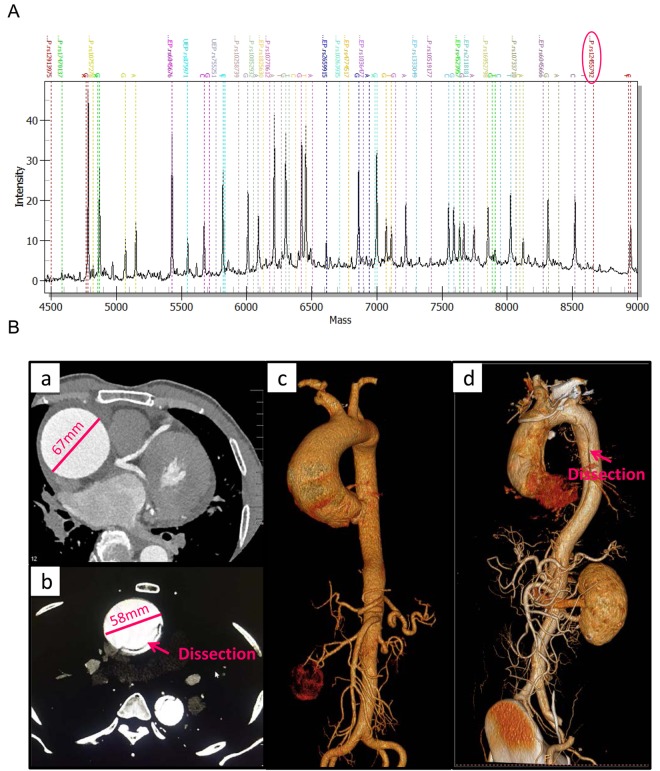
**The MALDI-TOF MS spectrum of candidate SNPs and large vessels CT angiography for clinical features of TAAD patients.** (**A**) The MALDI-TOF MS spectrum of candidate SNPs. Genotypes of SNPs are determined by plotting peak intensity (y-axis) against mass (Da) (x-axis). The spectrum of rs12455792 is indicated by a circle. (**B**) Large vessels CT angiography and 3D scanning images for clinical features of thoracic aortic aneurysm (**a**, **c**) and dissection (**b**, **d**) patients.

The demographic characteristics and clinical features of the study participants in screening and validation cohort were listed in Supplementary Table 1. Data of 400 healthy controls were documented in our previous study [19]. The candidate 20 SNPs were genotyped in 202 TAAD patients and 400 healthy controls using MALDI-TOF MS (Fig. 1A). Large vessels CT angiography and 3D scanning images for thoracic aortic aneurysm and dissection patients were shown in Fig. 1B.

The discriminations of genotype distribution for 20 candidate SNPs between cases and healthy controls were statistical analyzed using multiple regression (Supplementary Table 2). Genotype frequencies of all the SNPs in healthy controls were conformed to Hardy-Weinberg equilibrium (HWE) (*P*>0.05). Of the 20 SNPs, rs12913975, rs10757278, rs10770612, rs10733710 and rs12455792 were significantly associated with increased TAAD susceptibility, with ORs of 1.489 (*P*=0.012), 2.006 (*P*=0.005), 1.767 (*P*=0.003), 1.412 (*P*=0.005), 1.585 (*P*=0.011) adjusting for age and gender, respectively. Among them, the functional SNP rs12455792 C>T variant was correlated with increased aortic diameter of TAAD patients (r=0.164, *P*=0.020, positive correlation in screening data), yielding the most significant results (Supplementary Table 3). rs12455792 was bioinformatical predicted as a proximal transcription regulatory loci, i.e. it was a functional SNP. And we have proved that rs1245792 variant reduced the expression of *SMAD4* [19]. Collectively, we further investigated the relationship between rs12455792 and pathological progression of TAAD.

### rs12455792 C>T variant enhanced both canonical and non-canonical TGF-β signaling transduction in HASMCs

**Figure 2:**
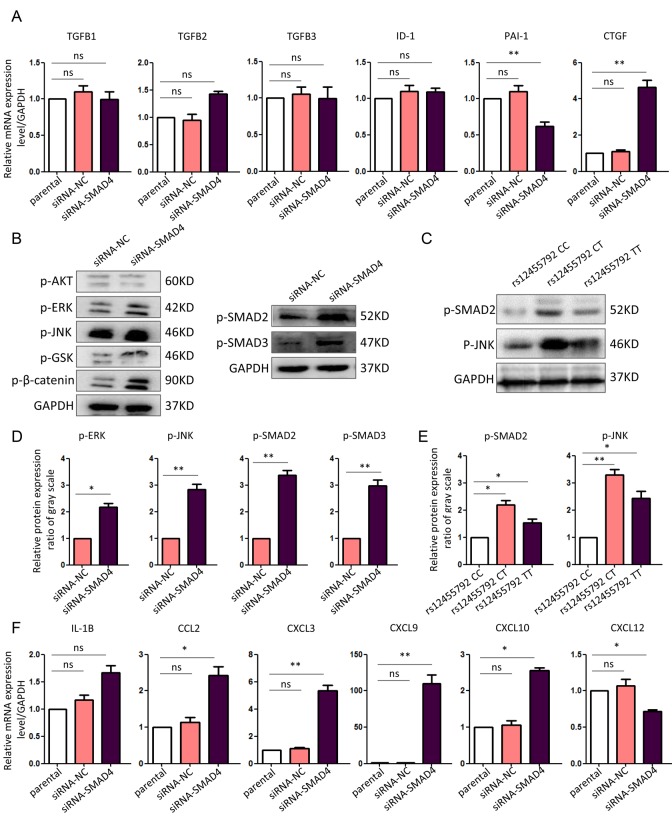
**Enhanced canonical and non-canonical TGF-β signaling in *SMAD4*-interfered HASMCs.** (**A**) Real-time PCR assay for the expression of canonical and non-canonical TGF-β signaling molecules in different HASMCs. (**B**) Western blot assay for the expression of canonical and non-canonical TGF-β signaling mediators in HASMCs transfected with siRNA-NC or siRNA-SMAD4. (**C**) Western blot assay for the expression of canonical and non-canonical TGF-β signaling mediators in HASMCs carrying rs12455792 CC, CT or TT genotypes. (**D**, **E**) Quantitative analysis for **B** and **C**, respectively. (**F**) Real-time PCR assay for the expression of essential chemotactic factors in different HASMCs. The experiments were repeated three times. Data were presented as mean ± SD. *P<0.05, **P<0.01, ns: not significant.

To determine the TGF-β signaling changes with rs12455792 C>T variant in HASMCs, we isolated normal human thoracic aortic SMCs and cultured them to passage 3 in SMC special medium. Since we have proved that rs12455792 variant suppressed *SMAD4* expression, siRNA-*SMAD4* were transfected into HASMCs to mimic the impact of rs12455792 variant genotype. Real-time PCR was used to detect the transcription of canonical ligands and downstream targets in TGF-β signaling in HASMCs transfected with siRNA-NC or siRNA-*SMAD4*. We found there was no change for the expression of TGFB1, TGFB2, TGFB3 and ID-1 between control and *SMAD4* low-expression group. But the expression of PAI-1 was obviously reduced in *SMAD4* low-expression group. PAI-1 was a direct target of canonical TGF-β pathway. Compared to control, we also found an obvious increase for the expression of CTGF in *SMAD4* low-expression group (Fig. 2A). This increase was offset with addition of canonical and non-canonical TGF-β signaling mediator inhibitors (Supplementary Figure 2). CTGF, an essential regulator in fibronectin assembly and vascular remodeling, is a well-known factor in TGF-β responsiveness.

Furthermore, we examined the activation of intracellular mediators in canonical or non-canonical TGF-β signaling. The results revealed an enhanced activation of SMAD2, SMAD3, JNK and ERK in *SMAD4* low-expression group (Fig. 2B, D). We also demonstrated the increased activation of SMAD2, and JNK in HASMCs carrying rs12455792 variant genotypes without gene dosage effect, compared to that with wild type genotype (Fig. 2C, E). These data confirmed that both canonical and non-canonical TGF-β signaling responsiveness elevated by *SMAD4* low-expression or rs12455792 variant in HASMCs.

### rs12455792 C>T variant leads to MØ recruitment accompanied by the increased expression of chemotactic factors

**Figure 3:**
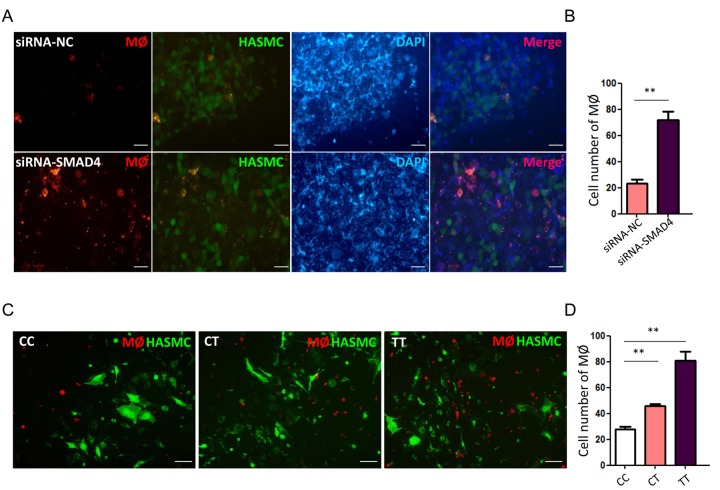
**rs12455792 C>T variant promote the chemotaxis of MØ to HASMCs**. (**A**) Representative fluorescence images of MØ migrating to HASMCs transfected with siRNA-NC or siRNA-SMAD4 in transwell assay. (**B**) The quantitative analysis of MØ cell numbers in different groups, n=5/group. (**C**) Representative fluorescence images of MØ migrating to HASMCs carrying rs12455792 CC, CT or TT genotypes in transwell assay. (**D**) The quantitative analysis of MØ cell numbers in different groups, n=5/group. Red: Dil-prestained MØ; Green: Dio-prestained HASMCs; Blue: DAPI. Data are means ± SD. **P<0.01, ns: not significant. Scale bar: 50μm.

Next, we investigated whether TGF-β signal interfering in HASMCs could directly trigger MØ recruitment. First, we checked the alterations for chemotactic factors in HASMCs transfected with siRNA-*SMAD4*. The real-time PCR data declared a significant upregulation of numerous chemokines, including CCL2, CXCL3, CXCL9, CXCL10 and CXCL12 in siRNA-*SMAD4* group (Fig. 2F). Among these CCL and CXCL family members, CCL2 and CXCL12 have been previously reported to facilitate monocyte chemotactic activity and be involved in vascular inflammation [20]. Therefore, we further detected MØ chemotaxis status with *SMAD*4-low expression and rs12455792 variant. Before functional assay, HASMCs were identified with over 95% SM22α immunostained and MØ was successfully induced from THP-1 human monocyte cell line by 100ng/ml PMA. The transwell migration assays verified an evident stimulating role of the *SMAD4*-silenced HASMCs on MØ chemotaxis, in compliance with the excessive CCL2 and CXCL12 secretion from the *SMAD4*-silenced HASMCs (Fig. 3A-B). Similarly, we observed rs12455792 C>T variant strikingly promoted MØ migrating to HASMCs (Fig. 3C-D).

### rs12455792 C>T variant facilitates MØ infiltration in human TAAD specimens

**Figure 4:**
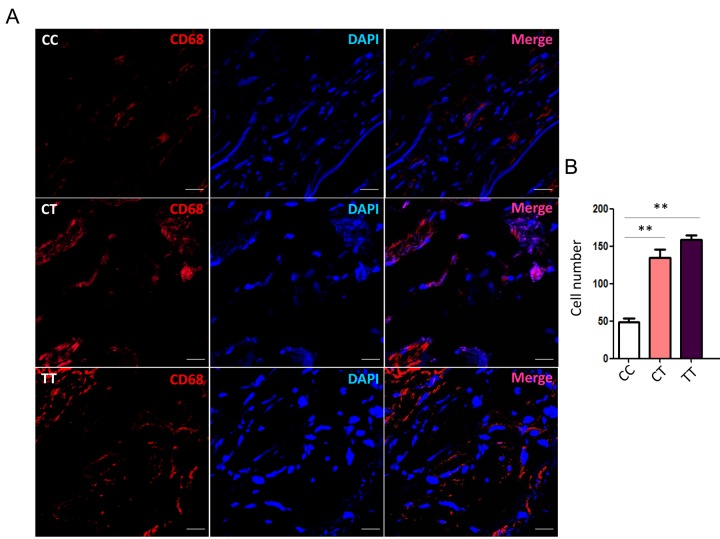
**rs12455792 C>T variant promotes MØ infiltration in human thoracic aorta specimens.** (**A**) Representative confocal-microscopy graphs of immunostaining for CD86 in specimens from thoracic aortic aneurysm patients with rs12455792 CC, CT or TT genotypes. Red: CD68; Blue: DAPI. (**B**) The quantitative analysis of MØ cell numbers in different groups, n=8/group. Data are means ± SD. **P<0.01, ns: not significant. Scale bar: 100μm.

**Figure 5:**
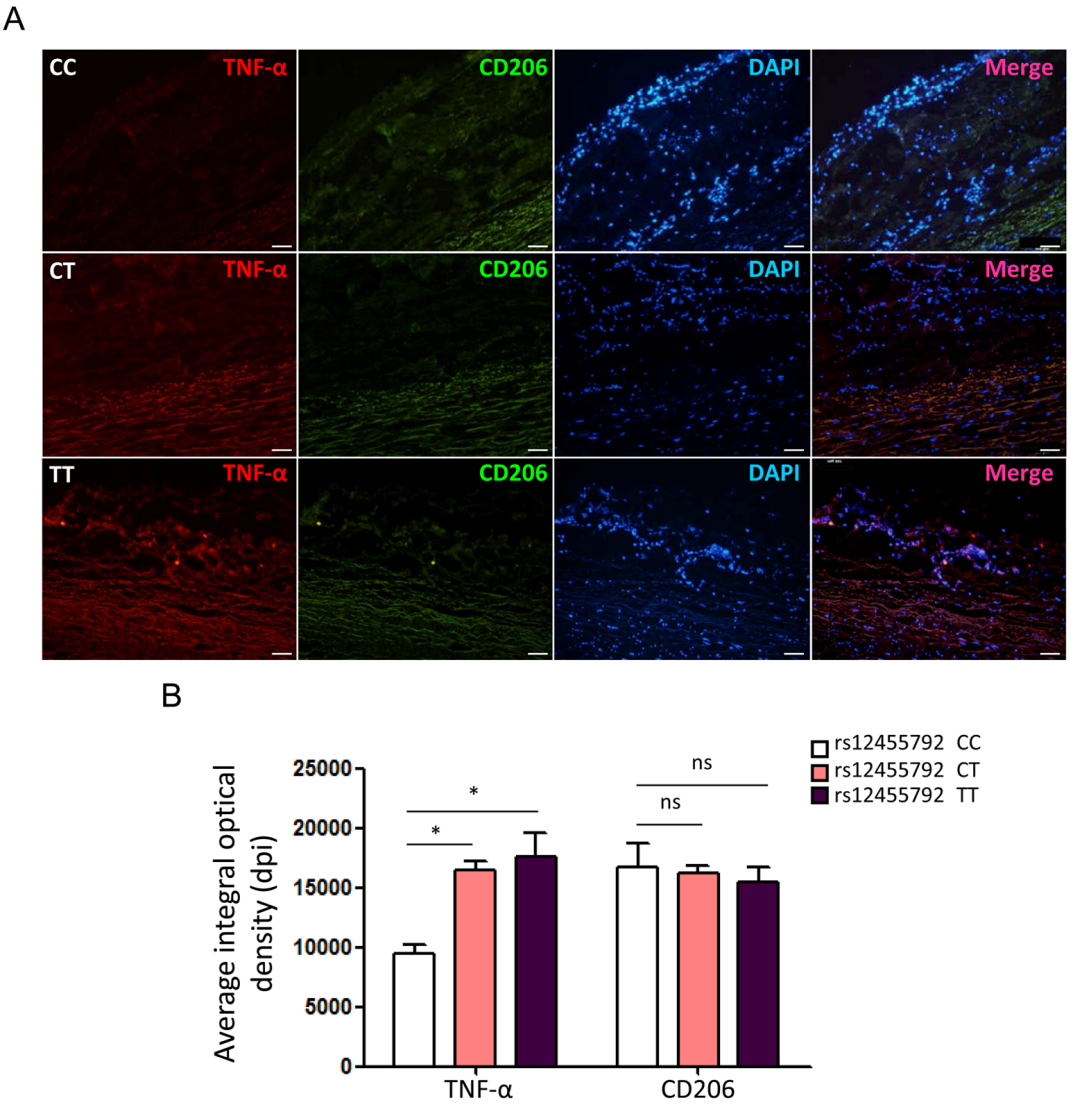
**rs12455792 C>T variant enhances TNF-α but not CD206 expression in human thoracic aorta specimens**. (**A**) Immunofluorescence staining for TNF-α and CD206 in specimens from thoracic aortic aneurysm patients with rs12455792 CC, CT or TT genotypes. Red: TNF-α; Green: CD206; Blue: DAPI. (**B**) The plot of average integral optical density for TNF-α and CD206 expression in different groups, n=8/group. Data are means ± SD. *P<0.05, **P<0.01, ns: not significant. Scale bar: 100μm.

To evaluate the actual phenomena of MØ infiltration in human thoracic aorta specimens from TAAD patients with different genotype, we examined immunofluorescence staining for CD68 with frozen sections of specimens under a laser scanning confocal microscopy. The number of CD68+ cells in rs12455792 CT and TT group was significantly higher than that in CC group (Fig. 4A-B). This suggested that rs12455792 C>T variant notably facilitated MØ infiltration in human TAAD specimens. Furthermore, we detected the expression of pro-inflammatory marker TNF-α (M1 type MØ) and anti-inflammatory marker CD206 (M2 type MØ) in human TAAD specimens with different genotype. The average integral optical density of TNF-α in CT or TT group was markedly increased, compared to that in CC group (Fig. 5A-B). rs12455792 C>T variant elevated M1 type MØ marker expression, indicating that the variant led to an activated inflammatory circumstance initiated via MØ polarization. But there was no significant change for CD206 expression with rs12455792 variant.

### SMAD4 knockdown facilitates the progression of TAAD in animal model

**Figure 6:**
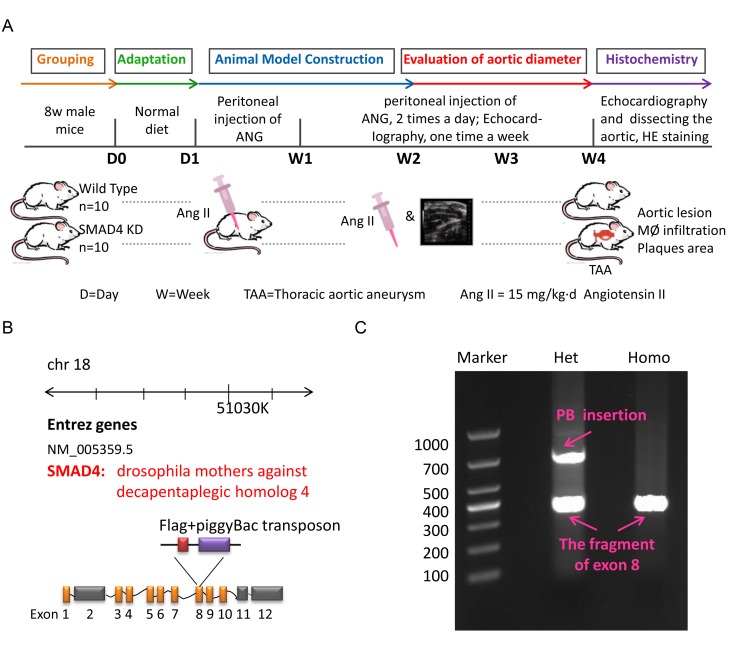
**The construction of TAA animal model.** (**A**) The flow diagram of modeling process for TAA mouse in Wild Type and SMAD4 KD group; (**B**) The schematic diagram for generation of SMAD4 KD mice; (**C**) The DNA electrophoretogram of identification for Wild Type mice and SMAD4 KD mice.

**Figure 7:**
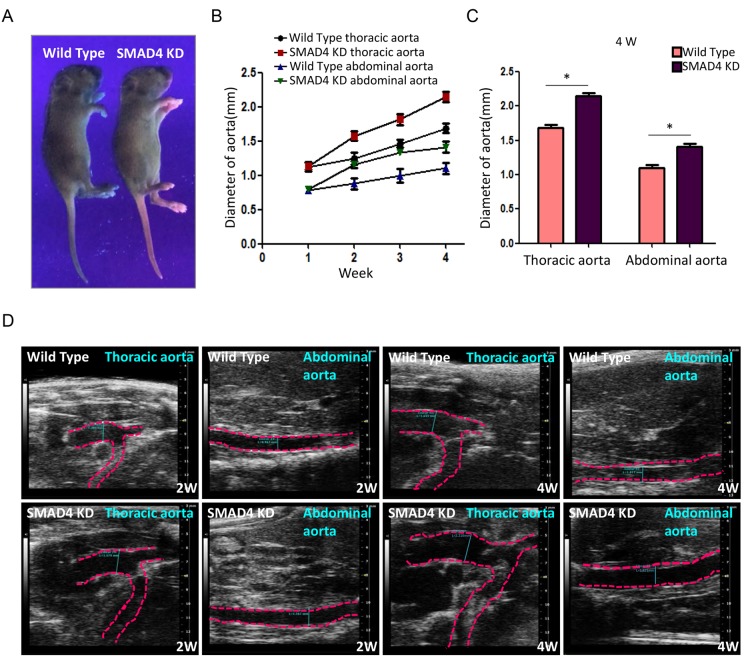
**Repression of *SMAD4* leads to dilatation of both thoracic aorta and abdominal aorta.** (**A**) Representative holistic view of Wild Type and SMAD4 KD mice under ultraviolet. The red fluorescence is observed in the naked skin of SMAD4 KD mice. (**B**) The polygram for time-dependent aorta dilatation in different groups; (**C**) The quantitative analysis for diameter of aortas among different groups at 4W after modeling; (**D**) Representative echocardiography images of aorta diameter in Wild Type and SMAD4 KD group at 2W or 4W, n=5/group. *: *P*<0.05. The data were presented as mean ± SD.

**Figure 8:**
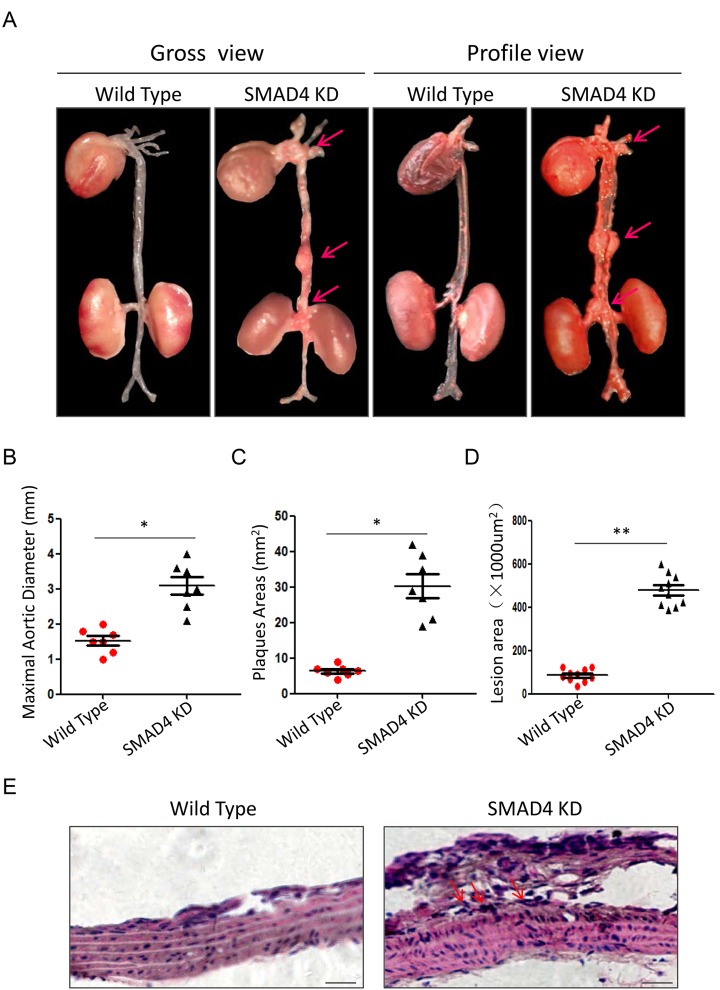
**Repression of *SMAD4* promotes the formation of TAAD.** (**A**) The gross view and profile view of aortas from Wild Type and SMAD4 KD mouse models. Quantitative analysis of maximal aortic diameter (**B**), plaques area (**C**) and lesion area (**D**) between different groups, n=5/group; (**E**) Representative graphs of HE staining for thoracic aortas from Wild Type and SMAD4 KD mouse models. Data are means ± SD. *P<0.05, **P<0.01. Scale bars: 100μm.

To mimic the impact of rs12455792 - *SMAD4* low expression generally, we constructed the *SMAD4* KD mouse and detected the associated pathological progress of TAAD. Animal model was constructed with peritoneal injection of 15mg/kg.d AngII for 4w. The procedure was shown in Fig. 6A. The *SMAD4* KD mouse was a heterozygote with one chromosome inserted by red fluorescence protein (RFP) flag and piggyBac (PB) transposon in exon 8 of *SMAD4* (Fig. 6B). It was identified by electrophoresis of mouse tail DNA (Fig. 6C). Applying ultraviolet transmission, the red fluorescence of inserted RFP flag was observed in the naked skin of *SMAD4* KD mice (Fig. 7A). With AngII administration for 4w, we found *SMAD4* knockdown strikingly contributed to the progressive dilatation of both thoracic aortas and abdominal aortas using echocardiography (from 1w to 4w) (Fig. 7B-D). Moreover, we separated the aortas in different groups and stained them by oil-red (Fig. 8A). As the aneurysm developed, *SMAD4* KD mice displayed typical pathological performances of the inherited predispose to TAAD, including aortic diameter increase, plaque accumulation, aortic wall thickening, monocytes infiltration and medial degeneration characterized by destructive vascular matrix remodeling (Fig. 8B-E). Notably, histological analysis of the thoracic aortic wall in *SMAD4* KD mice revealed that aortic dissection rapidly developed, accompanied with erythrocyte extravasation into the media lesion region and intense inflammatory cell infiltration (Fig. 8D-E).

## DISCUSSION

In the present study, we investigated the effects of 20 TAAD risk- related polymorphisms screened by GWAS and OMIM database. There was 5 significant SNP were identified using MALDI-TOF MS in 202 patients and 400 controls, rs12913975 (*SMAD6*), rs10757278 (*CDKN2B-AS1*), rs10770612 (*LINC02398*), rs10733710 (*TGFBR1*) and rs12455792 (*SMAD4*). Among them, rs12455792 was most associated with thoracic aorta dilatation and was chosen for further analysis. Functional experiments demonstrated that rs12455792 C>T polymorphism activated TGF-β signaling, enhanced MØ recruitment and M1 type inflammatory response, thus facilitated vascular remodeling and pathological progress of TAAD. To the best of our knowledge, this is the first study to demonstrate potential impact of rs12455792 on TGF-β signalling, MØ infiltration and pathogenesis of TAAD.

Our case-control study revealed a significant association between subjects carrying at least one T allele (CT, TT) of rs12455792 in *SMAD4* promoter with 1.59 fold increased risk of TAAD (Supplementary Table 2). In bioinformatical analysis, rs12455792 was an eQTL, located at -650C of *SMAD4* gene, a transcription factor binding site with proximal transcription regulatory potential. Our further experiments confirmed that rs12455792 C>T change reduced transcriptional activity and *SMAD4* expression [19]. Numerous studies suggested that *SMAD4*, the only one co-smad molecule in TGF-β/smad signaling, was essential in MØ infiltration and development of aortopathy [11-13,15]. *SMAD4* mutations have been identified as a genetic cause of hereditary vascular malformation syndrome and aortopathy [12,13]. However, the potential mechanism between rs12455792 variant, MØ infiltration, vascular remodeling and pathological progression of TAAD remained unclear. Thus we examined the TGF-β signaling change and MØ chemotaxis with rs12455792 variant.

In detection for TGF-β signaling molecules, there was an enhanced canonical and non-canonical signaling activation in *SMAD4* low-expression or rs12455792 variant groups. Activation of TGF-β signaling always contribute to uncontrolled cell growth and inflammatory response, leading to vascular disorders such as aneurysm [6]. The regulation of TGF-β signaling pathway is essential in the maintenance of vascular wall homeostasis. It plays a pivotal role in the synthesis and degradation of the extracellular matrix. Studies have confirmed the excessive activation of TGF-β signaling in the aortic wall of thoracic aortic aneurysm patients [21,22]. TGF-β signaling stimuli always activates the downstream molecules-SMAD2/3 by binding it to SMAD anchor for receptor activation (SARA), forming a SMAD-SARA complex. Then it is recognized by TGF-β receptor. SMAD2/3 are transported to the nucleus after phosphorylation. In this process, SMAD4 acts as a synergistic factor to form a stable SMAD2/3/4 complex, and assistances R-SMAD to regulate downstream gene expression [23]. Of note, we demonstrated that rs12455792 C>T variant elevated the phosphorylation of SMAD2 in canonical TGF-β signaling, and JNK in non-canonical TGF-β signaling, indicating that the change of rs12455792 participated in the abnormal activation of TGF-β signaling.

MØ was crucial mediators for the progression of aortic aneurysms and dissection, presumably through secreting many inflammatory factor to directly damage vascular matrix [24,25]. We speculated that rs12455792 related TGF-β signal interfering in HASMCs could directly trigger MØ recruitment. Real-time PCR data revealed a significant upregulation of numerous chemokines with *SMAD4* low-expression (Fig. 2F). Among these factors, CCL2 and CXCL12 have been previously reported to facilitate monocyte chemotactic activity and be involved in vascular inflammation [20]. Next, transwell assays verified an evident stimulating role of the *SMAD4*-silenced HASMCs on MØ chemotaxis, in compliance with the excessive CCL2 and CXCL12 secretion from the *SMAD4*-silenced HASMCs (Fig. 3A-B). Similarly, we observed rs12455792 C>T variant strikingly promoted MØ migrating to HASMCs (Fig. 3C-D). In human TAAD specimens, we also observed that rs12455792 C>T variant notably facilitated MØ infiltration (Fig. 4). The expression of pro-inflammatory marker TNF-α (M1 type MØ) in human TAAD specimens with CT or TT genotype was markedly higher than that of CC genotypes (Fig. 5). rs12455792 C>T variant promoted the activation of inflammatory circumstance, which contributed much to aggressiveness of TAAD. Our evidences are first to clarify a stimulating role of HASMCs with rs12455792 related TGF-β signaling disorder in facilitating thoracic aorta wall inflammation, which ultimately contribute to the progression of TAAD.

To address the role of rs12455792 on vascular remodeling in TAAD, we constructed *SMAD4* KD mouse to mimic the impact of rs12455792. With AngII administration for 4w, we found *SMAD4* knockdown strikingly contributed to the progressive dilatation of thoracic aortas using echocardiography (Fig. 7B-D). As the aneurysm developed, *SMAD4* KD mice displayed typical pathological performances of the inherited predispose to TAAD, including aortic diameter increase, plaque accumulation, aortic wall thickening, monocytes infiltration and medial degeneration characterized by destructive vascular matrix remodeling (Fig. 8B-E). Notably, histochemistry analysis of the thoracic aortic wall in *SMAD4* KD mice revealed that aortic dissection rapidly developed, accompanied with erythrocyte extravasation and intense inflammatory cell infiltration (Fig. 8D-E). These phenomena suggested that *SMAD4* low-expression, similar to the impact of rs12455792, promoted the pathological remodeling of the aorta and trigger the formation of TAAD *in vivo*. Our data was consistent with the report that *SMAD4* deletion largely recapitulates vascular phenotypes of LDS patients, with widespread aneurysm and early dissection [15].

**Figure 9:**
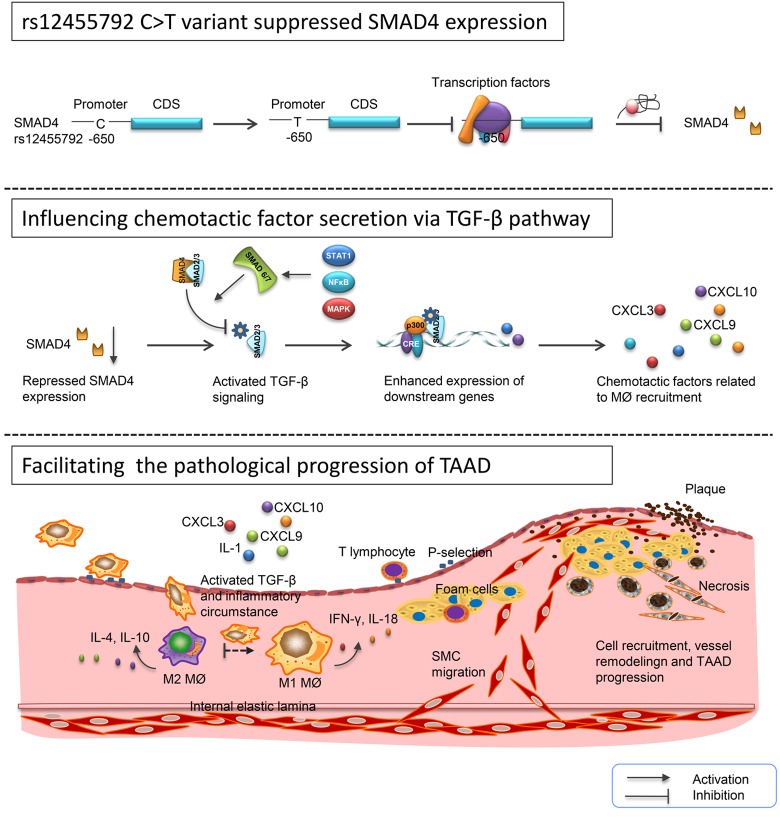
**Schematic diagram for the potential role of rs12455792 variant in pathological progression of TAAD.** rs12455792 C>T variant might suppress *SMAD4* expression by affecting the binding of transcription factors to the promoter of this gene, thus activate TGF-β signaling and chemotactic factors production, and further facilitate the MØ recruitment, vessel remodeling and pathological progression of TAAD.

In conclusion, we identified 5 significant SNPs on the basis of GWAS-based TAAD reports in 602 subjects. Among them, rs12455792 was correlated with aortic diameter of TAAD patients. This variant enhanced MØ recruitment, M1 type inflammatory response via activating TGF-β signaling, further promoted vascular remodeling and TAAD progression (Fig. 9). In mouse model, we verified that *SMAD4* knock down facilitated the pathological development of TAAD. Our study elucidated the potential mechanism for differences in susceptibility and prognosis of TAAD between patients with CC, CT or TT genotypes. These novel findings may shed new light on the role of rs12455792 and *SMAD4* in pathogenesis of TAAD and provide a predictive marker for optimizing clinical trial design and individualizing therapeutic plans.

## CONCLUSIONS

1. The variant rs12455792 in *SMAD4* gene significantly elevated canonical and non-canonical TGF-β signaling.

2. The variant rs12455792 reduced *SMAD4* expression and influenced its effects on chemotactic factors secretion, MØ recruitment.

3. rs12455792 C>T variant promoted MØ infiltration and M1 type inflammatory response in human aneurysm specimens, further influenced vascular remodeling and pathological progression of TAAD.

Understanding the genetic features of thoracic aortic aneurysm and dissection can lead to precision surgery strategy. In this study, we demonstrated that rs12455792 in *SMAD4* gene, which reduced transcription activity and *SMAD4* expression, enhanced MØ recruitment, M1 type inflammatory response via activating TGF-β signaling, further promoted vascular remodeling and TAAD progression. These findings may provide a predictive marker for optimizing clinical trial design of TAAD.

## MATERIALS AND METHODS

### Subjects

All experiments referring human or animal samples were approved by Institutional Review Board (IRB), the first affiliated hospital of Soochow University (Jan. 2010 - Dec. 2016). All subjects were chosen from Han Chinese population of eastern China. 202 cases of screening cohort, 227 cases of validation cohort and 400 healthy controls were included in this study. They have signed the informed consent. The inclusion criteria of sporadic TAAD patients was described in previous report [19]. Controls were frequency matched via age and gender to cases. The aorta status of patients were measured by echocardiography, angiography, CT or MRI. The demographic and clinicopathological information were documented in patients' interview. Fresh thoracic aorta tissues around focus were collected from 39 TAAD patients receiving the Bentall procedures in Department of Cardiovascular Surgery, the first affiliated hospital of Soochow University.

### SNP selecting and genotyping

We selected 30 SNPs essential in pathogenesis of TAAD by analyzing GWAS-based TAAD reports [16-18], OMIM database (http://www.omim.org/) and Han Chinese data from 1000 Genome Project resources (http://www.1000genomes.org). Among them, 10 SNPs were difficult to detected using matrix-assisted laser desorption ionization time-of-flight mass spectrometry (MALDI-TOF MS) and excluded, by virtue of homologous interference, hairpin structure, short intervals and so on. All the candidate SNPs meet the following criteria: (1) the minor allele frequency (MAF)>0.05; (2) r^2^>0.80 for each paired SNPs. Functinal SNPs were bioinformatical analyzed using online tools- rsnp (http://rsnp.psych.ac.cn/) and snpinfo (http://snpinfo.niehs.nih.gov/cgi-bin/snpinfo/snpfunc.cgi).

Candidate SNPs were genotyped using MALDI-TOF MS as previously described [19]. Briefly, The PCR product was dispensed into a 384-format SpectroCHIP and the MALDI-TOF MS assay was performed on a MassARRAY Compact Analyzer (Sequenom, San Diego, CA, USA). Amplification and single-base extension primers in multiple PCR were synthesized by Benegene (Benegene Biotechnology, Shanghai, China) and all the sequences were listed in Supplementary Table 4. Genotype calling was carried out with MassARRAY RT package 3.0. Genotyping quality was evaluated by Sanger sequencing for ~15% randomly chosen samples, finally generating a 100% concordance. The success rate of SNP genotyping was >99%.

### Animal models

The *SMAD4* knock-down (KD) mouse were designed and constructed by Biogle (Biogle Inc., Hangzhou, China). All mice were maintained with C57BL/6J genetic background. Experiments regarding to animal model were performed according to institutional guidelines for laboratory animals. To induce aneurysm formation of TAA model, we chose 10-12w old mice and intraperitoneal injected with Angiotensin II (Ang II) (Sangon Inc., Shanghai, China) (15mg/kg.d, n =10 per group). All experiments were conducted using both male and female mice. Controls were sex-matched littermates. Four weeks later, mice were sacrificed, and thoracic aortas were histochemistry stained and evaluated.

### Echocardiography

Mice were anesthetized with 5% isoflurane by Small Animal Anesthesia Ventilator System (RWD Life Science Inc,. Shenzhen, China). The thoracic aortic diameter was evaluated using a Vevo2100 cardiovascular ultrasound system with MS400 (30mHz) microprobe (VisualSonics Inc., Toronto, Canada). Echocardiographic detection was performed in B mode. Each experiment was repeated at three times from 10 mice per group.

### Cell culture

Primary patients human aortic smooth muscle cells (HASMCs) were isolated from thoracic aorta of TAAD patients who received Bentall procedures. Normal HASMCs was obtained from aorta from patients during aortic valve replacement. In brief, the media of fresh thoracic aortas were tore off and cut into 1mm^2^ pieces, then cultured in 10cm dishes with human SMC medium (Sciencell, San Diego, CA, USA). Three days after primary culture, HASMCs were spread out from aortic tissues. With growth to 80% confluence, cells were digested by 0.25% trypsin-EDTA and passaged. In the experiments, HASMCs were used in passage 3-8 with at least 95% purity.

### Real-time PCR and western blotting

Real-time PCR was performed on an ABI StepOnePlus™ Real-Time PCR System (ABI, Carlsbad CA, USA). The primers used for amplification of chemotactic factors and TGF-β molecules were listed in Supplementary Table 5. In western blotting assay, proteins were extracted from HASMCs or human thoracic aorta tissues and subjected to immunoblotting with rabbit polyclonal antibodies against p-ERK、p-JNK、p-GSK, mouse monoclonal antibodies against pSMAD2、pSMAD3 (1:1000, Santacruz, California, USA). GAPDH was used as internal control. Gel-Pro analyzer 4.0 software package was used to analysis the grey scales of target proteins, which were standardized against that of GAPDH (Media Cybernetics, Silver Spring, MD, USA).

### Chemotatic assay

MØ was derived from THP-1 monocytes with 100ng/ml PMA induction for 48h. Cell chemotaxis were measured using 24-well Transwell® units (8.0 μm pore, Costar Corning, NY, USA). One hour before experiment, MØ was stained with Dil (red, Beyotime, Shanghai, China), a bioactive fluorescence probe. HASMCs were stained with Dio (green, Beyotime, Shanghai, China). Then, MØ (5×10^3^) in 100 μl medium was added into each upper insert, and HASMCs in different group (5×10^4^) in 500 μl medium were added into lower chamber. The plate were incubated at 37 °C, 5% CO_2_ for 24h. To examine MØ chemotaxic-migrating to lower chamber, cells was fixed by methanol for 10 minutes, washed, and stained with DAPI. Finally, the images of chmotaxic-migrated MØ were captured with an inversion fluorescence microscope (Olympus IX51, Tokyo, Japan). MØ was quantified by counting 10 independent visual fields per group via Image Pro Plus 6.0 software (Media Cybernetics, Silver Spring, MD, USA). Each assay was performed in triplicate.

### Quantification of aortic lesions

The aortic tissue of mouse was removed from the ascending aorta to the iliac artery branch and fixed with 4% paraformaldehyde overnight at 4°C. After fixation, the aortic intimal surface was exposed by a longitudinal cut through the aortic arch that extended down the whole length of the aortic tree. Then the aortic tissues were stained using Oil Red-O and images were captured. To quantify the phanerous lesions in intimal surface, mean lesion areas was analyzed by Image Pro Plus 6.0 software (Media Cybernetics, Silver Spring, MD, USA).

### Histological and immunocytochemical characterization of TAAD

To evaluate the pathological performances of TAAD, murine aortas were separated and fixed in the 4% paraformaldehyde after 4w of treatment. Serial paraffin cross section (5 μm) of murine thoracic aortas were prepared for histological analysis. Aorta sections were stained with eosin and hematoxylin for morphological assessment. Immunostaining of CD86 and polarization markers (TNF-α, CD206) (1:1000, Santacruz, California, USA) was performed to detect MØ infiltration in frozen sections of human thoracic aortas. The images of immunofluorescence were digitally captured on a Carl Zeiss LSM880 laser scanning confocal microscope (Carl zeiss, Jena, Germany). CD68+ Cell numbers or average integral optical density of CD206 and TNF-α in the aortic wall were quantitative analyzed from 10 samples per group using Image Pro Plus 6.0 software (Media Cybernetics, Silver Spring, MD, USA).

### Statistical analysis

Differences of the demographic and clinical features, and frequencies of genotypes in case-control study were tested by Student's *t* test (for continuous variables) or Chi-square test (for categorical variables). The comparisons of cell numbers, mean intergral optical density, lesion areas and so on were using Student's *t* test (between 2 groups) or One way ANOVA (between more than 2 groups). Hardy–Weinberg equilibrium (HWE) was evaluated using online analytical tools. For analyzing main effect of candidate SNPs, univariate or multivariate logistic regression models were performed to generate ORs and corresponding 95%CIs with adjustment by possible confounders. All statistical tests were two tailed and conducted using Statistical Program for Social Sciences (SPSS 18.0, Chicago, IL, USA) or R software(http://www.r-project.org/). *P* value <0.05 was considered statistically significant.

## Supplementary Material

Supplementary Figures

Supplementary Tables
