# Correction: A functional variant of *SMAD4* enhances macrophage recruitment and inflammatory response via TGF-β signal activation in Thoracic aortic aneurysm and dissection

**DOI:** 10.18632/aging.101812

**Published:** 2019-01-31

**Authors:** Ying Wang, Pei Yin, Yi-Huan Chen, Yun-Sheng Yu, Wen-Xue Ye, Hao-Yue Huang, Zhen-Chun Ji, Zhen-Ya Shen

**Affiliations:** 1 Department of Cardiovascular Surgery of the First Affiliated Hospital and Institute for Cardiovascular Science, Soochow University, Suzhou, Jiangsu, China; 2 Department of Thoracic Surgery, Taizhou People's Hospital, Taizhou, Jiangsu, China; * Equal contribution

Original article: Aging (Albany NY) 2018; 10: 3683-3701

**This article has been corrected:** The authors made mistake in Supplementary Table 1, the D-dimer, CRP data are same in screening cohort and validation cohort. The correct Supplementary Table 1 is provided below. The authors declare that this correction does not change the results or conclusions of this paper. The authors sincerely apologize for this error.

**Supplementary Table 1:** Demographics and clinical features of the TAAD patients involved in this study.

Item	Screening cohort n=202	Validation cohort n=227	*P*
Age	54.1±14.2	55.2±12.7	0.633
Men(%)	75.2	72.1	0.727
Hypertension(%)	40.1	37.3	0.423
Diabetes(%)	14.4	12.6	0.652
Smoking(%)	52.0	46.5	0.543
Body mass index (kg/m^2^) Thoracic aortic diameter(mm)	23.9±4.0 53.2±8.0	24.5±4.2 52.2±9.7	0.768 0.439
D-dimer(μg/ml)	7.66±2.81	7.82±3.98	0.185
CRP(μg/ml)	13.22±6.33	12.69±5.17	0.299
HDL-c	1.15±0.29	1.26±0.29	0.071
LDL-c	2.45±0.80	2.39±0.87	0.299

