# Supplementary material for: A functional variant of SMAD4 enhances macrophage recruitment and inflammatory response via TGF-β signal activation in Thoracic aortic aneurysm and dissection
Source: Aging (Albany NY). 2018 Dec 7;10(12):3683–701. doi: 10.18632/aging.101662 (PMC6326647; doi:10.18632/aging.101662)
Supplement: Supplementary Figures [file aging-10-101662-s001.pdf]

SUPPLEMENTARY FIGURES

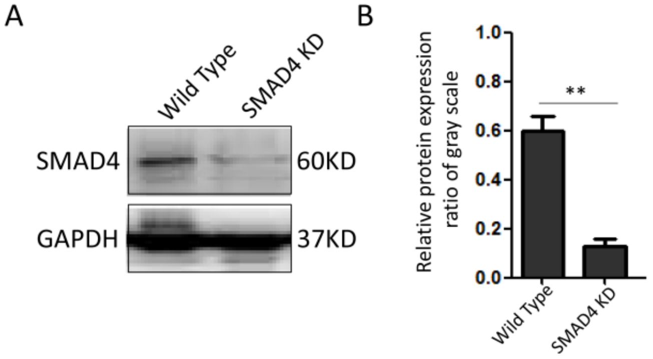

Supplementary Figure 1. Significantly reduced SMAD4 expression in *SMAD4*-KD mouse. (**A**) Western blot for SMAD4 expression in Wild Type and *SMAD4*-KD mouse. (**B**) Quantitative analysis for (**A**) Data were presented as mean ± SD. ***P*<0.01.

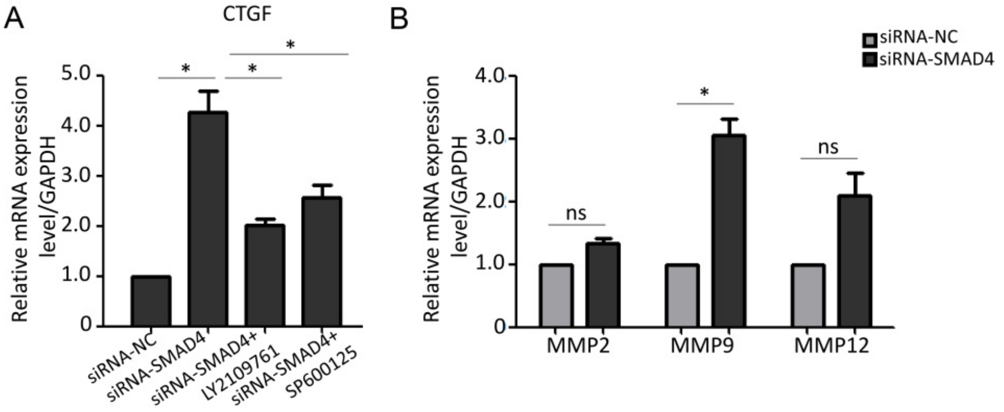

Supplementary Figure 2. CTGF and MMPs expression in different HASMCs. (**A**) qPCR detection for CTGF expression in HASMCs treated with siRNAs or SMAD2/JNK phosphorylation inhibitors. (**B**) qPCR detection for MMPs expression in HASMCs transfected with siRNA-NC or si-RNA-*SMAD4*. Data were presented as mean ± SD. **P*<0.05, ns: not significant.
