# Supplementary material for: A functional variant of SMAD4 enhances macrophage recruitment and inflammatory response via TGF-β signal activation in Thoracic aortic aneurysm and dissection
Source: Aging (Albany NY). 2018 Dec 7;10(12):3683–701. doi: 10.18632/aging.101662 (PMC6326647; doi:10.18632/aging.101662)
Supplement: Supplementary Tables [file aging-10-101662-s002.pdf]

SUPPLEMENTARY TABLES

Supplementary Table 1. Demographics and clinical features of the TAAD patients involved in this study.

Item	Screening cohort n=202	Validation cohort n=227	*P*
Age	54.1±14.2	55.2±12.7	0.633
Men(%)	75.2	72.1	0.727
Hypertension(%)	40.1	37.3	0.423
Diabetes(%)	14.4	12.6	0.652
Smoking(%)	52.0	46.5	0.543
Body mass index (kg/m^2)	23.9±4.0	24.5±4.2	0.768
Thoracic aortic diameter(mm)	53.2±8.0	52.2±9.7	0.439
D-dimer(μg/ml)	7.82±3.98	7.82±3.98	0.185
CRP(μg/ml)	13.22±6.33	13.22±6.33	0.299
HDL-c	1.15±0.29	1.26±0.29	0.071
LDL-c	2.45±0.80	2.39±0.87	0.299

Supplementary Table 2. Logistic regression analysis of associations between candidate SNPs and TAAD risk.

Loci (Gene)	HWE, *P*	Crude OR (95%CI)	*P*[a]	Adjusted OR (95%CI)	*P*[b]
rs12913975(SMAD6) G>A	0.189	1.500(1.113-2.022)	**0.009**	1.489(1.101-2.009)	**0.012**
rs17470137(CCDC100) G>A	0.072	1.119(0.102-12.239)	1.000	1.108(0.097-12.230)	1.000
rs10757278(CDKN2B-AS1) A>G	0.349	2.020(1.353-3.016)	**0.001**	2.006(1.342-3.002)	**0.005**
rs6045676(PDYN-AS1) C>G	0.391	1.057(0.811-1.377)	0.743	1.046(0.800-1.362)	0.855
rs875971(CRCP) T>C	0.472	0.730(0.613-1.068)	0.112	0.722(0.601-1.052)	0.236
rs755251(FBN1) A>G	0.563	1.001(0.860-1.164)	1.000	0.987(0.853-1.159)	1.000
rs10258739(TYW1) A>T	0.411	1.027(0.874-1.206)	0.832	1.015(0.869-1.201)	0.952
rs10852932(SMG6) T>G	0.237	0.985(0.653-1.485)	0.941	0.976(0.642-1.471)	0.989
rs1825630(FHIT) C>T	0.089	1.020(0.911-1.143)	0.809	1.009(0.893-1.131)	0.925
rs10770612(LINC02398) A>G	0.152	1.773(1.252-2.510)	**0.001**	1.767(1.245-2.497)	**0.003**
rs2659915(RABGEF1) G>A	0.723	0.901(0.717-1.132)	0.407	0.893(0.708-1.121)	0.519
rs10263935(KCTD7) G>A	0.171	0.938(0.750-1.174)	0.605	0.938(0.750-1.174)	0.753
rs4774517(FBN1) G>T	0.526	0.994(0.864-1.143)	1.000	0.987(0.859-1.137)	1.000
rs1036477(FBN1) A>G	0.109	0.959(0.775-1.188)	0.755	0.952(0.769-1.181)	0.829
rs1333049(CDKN2B-AS1) G>C	0.652	0.714(0.563-1.119)	0.543	0.709(0.558-1.112)	0.597
rs10519177(FBN1) A>G	0.328	1.005(0.875-1.153)	1.000	1.001(0.870-1.149)	1.000
rs2118181(FBN1) T>C	0.419	0.959(0.775-1.188)	0.755	0.952(0.769-1.181)	0.837
rs10733710(TGFBR1) G>A	0.563	1.431(1.143-1.792)	**0.002**	1.412(1.140-1.785)	**0.005**
rs12455792(SMAD4) T>C	0.936	1.591(1.093-2.312)	**0.009**	1.583(1.091-2.304)	**0.011**
rs6045666(PDYN-AS1) C>T	0.723	1.048(0.805-1.362)	0.747	1.043(0.802-1.360)	0.823

CI, confidence interval; OR, odds ratio; The results were in bold, if *P* <0.05.
[a] Chi-square test for genotype distributions between cases and controls. [b] Adjusted for age, sex, smoking status in logistic regress models.

Supplementary Table 3. The association between significant SNPs and thoracic aortic diameter in the study.

Significant SNPs	D-Thoracic aortic diameter (mm)			
Genotypes	Screening data (n=202)		Validation data (n=227)	
	D≤40/40<D≤50/D>50	P^a	D≤40/40<D≤50/D>50	P^b
rs10733710				
GG	44/49/26	0.477	51/51/28	0.501
GA+AA	36/30/17		41/37/20	
rs10757278				
AA	29/28/12	0.396	33/31/15	0.357
AG+GG	51/51/31		59/57/33	
rs12455792				
CC	29/17/8	**0.020**	34/22/12	**0.016**
CT+TT	51/62/35		57/66/36	
rs12913975				
GG	63/53/27	**0.046**	70/55/26	**0.042**
GA+AA	17/26/16		22/33/22	
rs1333049				
GG	31/41/19	0.387	38/46/23	0.452
GC+CC	49/38/24		54/42/25	

The significant *P* value were in bold.
MM/Mm/mm: Major homozygote/heterozygote/minor homozygote.
[a] For screening data. [b] For validation data.

Supplementary Table 4. Primers for PCR and single-base extension reaction in MALDI-TOF MS assay.

Loci (Gene)	Chr:position	Forward primer[a]	Reverse primer[a]	Extension primer	Amplicon size(bp)
rs12913975 (SMAD6)	15:66764823	linker-TCCTTCTTTGATCTCACGGC	linker-GGGTACTTGATGTCAGCAAC	TCCATCCAGCTAGCA	212
rs17470137 (CCDC100)	5:123195653	linker-TTCTTTGCTTACGCTGTAGG	linker-TCAGAATCGTCCCATCCAAG	CGCTGTAGGCTCAGT	145
rs10757278 (CDKN2B-AS1)	9:22124478	linker-AGTTGGAACTGAACTGAGGC	linker-ACTCTGTCTTGATTCTGCAT	TATTCTGCATCGCTGC	167
rs6045676 (PDYN-AS1)	20:1960525	linker-GAATTCATCACCAGCATACC	linker-TCAGACTCCCATCTGGTATC	CACCAGCATACCTGTATA	192
rs875971 (CRCP)	7:66152608	linker-AACAGGCTGAGGTTGTCAGG	linker-AAAGGCCGTGGAGGAAATCT	GGAGGAAATCTTTCAAGC	182
rs755251 (FBN1)	15:48519823	linker-ACTGGGATTAGATTTGGGAC	linker-CTCAGGCAATATTTAGTGGC	CATTTGCTACTTAGCACAG	101
rs10258739 (TYW1)	7:66597948	linker-GAGTGAATAATACCACCCTC	linker-CTGAGTTCACCACGAGTTTC	CCAATACCACCCTCATTTCA	95
rs10852932 (SMG6)	17:2240166	linker-GAGATGACTCTGTCCCTATC	linker-TCACAAGGCAGCCACTTTAG	CCCGCCACTTTAGTCAGTGA	107
rs1825630 (FHIT)	3:59917075	linker-ATGGACTCTGAGCCCATTGT	linker-GGGCTGGAGAGAACCATTAT	AAGAGATCCAAGTTCAACAA	229
rs10770612 (LINC02398)	12:20077705	linker-AGAGTCTAATCTGTTTGGGC	linker-ATCTCTGTGCCAATACCCTG	GGGGTTACCAATACAGGAAC	171
rs2659915 (RABGEF1)	7:66688114	linker-TGTTATTGCACTCCAGCCT	linker-TGCCTAACATATGGCCACTC	CCTTGCCACTCCTGATGTCATT	155
rs10263935 (KCTD7)	7:66631041	linker-TCTGAGCCCTTGATATACAC	linker-GCACCCACAGGACATGAAAA	CTCTAGGACCTACAGAATAAGC	91
rs4774517 (FBN1)	15:48467094	linker-ATGGGTGACAACTAGTGGAG	linker-TCAAAATAGGCCTCAGAAAG	GGACAACTAGTGGAGTCCTAAT	102
rs1036477 (FBN1)	15:48622729	linker-CCTACAGCCCAAAGATGGAC	linker-GTGGAAGTTGAAGGTATAGG	AGGTATAGGTAAAGATAAGAGT	201

rs1333049 (CDKN2B-AS1)	9:22125504	linker-ATGGTCACTACCCTACTGTC	linker-GCATACTTTTGTCAACACAG	ATACTAACCATATGATCAACAGTT	124
rs10519177 (FBN1)	15:48464998	linker-CTTATGCAGAGCACAAGGAG	linker-AGCCATGAGCCATGGTTTTC	GGTCGACATGGAGGAATCACACAA	178
rs2118181 (FBN1)	15:48623687	linker-TCATGCGGCCAATTCCTACA	linker-CAAGCAGCACATCCAAAGAG	GGAAAGACAATCATGACAAAATTCA	113
rs10733710 (TGFBR1)	9:99145142	linker-TGATGAAAGGTTGCCCTTCC	linker-ACCTATTAGACGATTGGCAC	GGGGAACATAGTAGGTGCCCTATAAA	97
rs12455792 (SMAD4)	18:51046270	linker-AGTGAGGGTCCAATTTCTCG	linker-GCTACTTCATACCTACTGAG	CTGAGATGGATATATCAAAAAGACAATA	106
rs6045666 (PDYN-AS1)	20:1959858	linker-GCTTGAAGGAGAAATAATGG	linker-GGGTTCACTGGGTTTCTTCA	ACTTTGCCAAATTTGATGTAAACAAAA	121

[a]linker=10-mer linker ACGTTGGATG placed at 5'-end of each primer.

Supplementary Table 5. Primers of TGF-β signaling molecules and chemotactic factors in real-time PCR assay.

Gene	Species	Primer sequence	Amplicon size(bp)
GAPDH	Human	5’-GAGCACAAGAGGAAGAGA-3’<br>5’-CACAGGGTACTTTATTGATGG-3’	167
IL1-B	Human	5’-CAGAAGTACCTGAGCTCGCC-3’<br>5’-AGATTCGTAGCTGGATGCCG-3’	153
CX3CL1	Human	5’-CGCGCAATCATCTTGGAGAC-3’<br>5’-AGGACCACAGACTCGTCCAT-3’	200
CXCL3	Human	5’-AACCGAAGTCATAGCCACACT-3’<br>5’-ATTTTCAGCTCTGGTAAGGGC-3’	192
CXCL9	Human	5’-TTTCCCGAAGGAAAAGGGCT-3’<br>5’-TCCACTAACCGACTTGGCTG-3’	227
CXCL10	Human	5’-CGCTGTACCTGCATCAGCAT-3’<br>5’-TGCATCGATTTTGCTCCCCT-3’	239
CXCL12	Human	5’-CTACAGATGCCCATGCCGAT-3’<br>5’-GTGGGTCTAGCGGAAAGTCC-3’	226
CXCL13	Human	5’-TGACTCTGCTAATGAGCCTGG-3’<br>5’-ATTCCCACGGGGCAAGATTT-3’	232
CCL2	Human	5’-TTCCCCTAGCTTTCCCCAGA-3’<br>5’-TCCCAGGGGTAGAACTGTGG-3’	204
CCL3	Human	5’-GCTCTCTGCAACCAGTTCTCT-3’<br>5’-CGGCTTCGCTTGGTTAGGAA-3’	164
CCL5	Human	5’-CAGTCGTCTTTGTCACCCGA-3’<br>5’-CGGGTGGGGTAGGATAGTGA-3’	194
TGFB1	Human	5’-CTGTCCAACATGATCGTGCG-3’<br>5’-TGACACAGAGATCCGCAGTC-3’	216
TGFB2	Human	5’-CGAAACTGTCTGCCCAGTTG-3’<br>5’-TCCGTTGTTCAGGCACTCTG-3’	239
TGFB3	Human	5’-GTGCCGTGAACTGGCTTCT-3’<br>5’-TCTTCATTGGCTGGGGTGTG-3’	209
CTGF	Human	5’-AAGGGCAAAAAGTGCATCCG-3’<br>5’-CTTCTTCATGACCTCGCCGT-3’	219
ID-1	Human	5’-AAACGTGCTGCTCTACGACA-3’<br>5’-AGGAACGCATGCCGCC-3’	256
PAI	Human	5’-AGAACCTGGGAATGACCGAC-3’<br>5’-TTGTGCCGGACCACAAAGAG-3’	223
